# What evidence exists on how biodiversity is affected by the adoption of carbon footprint-reducing agricultural practices? A systematic map

**DOI:** 10.1186/s13750-025-00372-7

**Published:** 2025-10-11

**Authors:** Stuart Rowlands, Julia Casperd, Michael R. F. Lee, Scott Kirby, Nicola Randall

**Affiliations:** Harper Adams University, Edgmond, Shropshire TF10 8NB UK

**Keywords:** Agroecosystem, Evidence synthesis, Farming, Greenhouse gas, Land use, Net zero

## Abstract

**Background:**

The global agriculture sector is expected to contribute towards carbon net zero by adopting interventions to reduce/offset greenhouse gas emissions and increase carbon sequestration/removal. Many of these interventions require change to land management and agriculturally associated habitats, subsequently impacting biodiversity. This relationship is important as the Convention on Biological Diversity has also pledged to reverse nature decline. To understand this relationship, a systematic map was developed to collate evidence relating to the impacts of carbon footprint reducing interventions on agriculturally associated biodiversity. This systematic map collated studies from temperate farming systems including northern Europe, North America and New Zealand.

**Methods:**

A protocol was published to define the methodology. Potentially relevant articles were identified by searching three academic databases using a predefined search string. Also, nine organisational websites were searched using key words. All potentially relevant articles were exported into EPPI-Reviewer-Web. Following deduplication, the remaining articles were screened at title and abstract level, partially with the aide of machine learning, before full text screening and extraction of metadata.

**Review findings:**

Screening began with 67,617 articles that ended with an evidence base of 820 primary research studies and 82 reviews. The evidence base includes studies from 1978 to April 2024, of which 81% were studies that lasted less than 5 years. Whilst microorganisms (*n* = 328), arthropods (*n* = 190), worms (*n* = 121) and plants (*n* = 118) were well represented in the evidence base, other groups such as birds (*n* = 32), gastropods (*n* = 16), mammals (*n* = 13), amphibians (*n* = 1) and reptiles (*n* = 1) were represented less well. The most studied interventions were to increase soil organic carbon through reduced tillage (*n* = 227) and cover cropping (*n* = 136). However, there were less than five studies in total for the following land management objectives: avoiding soil compaction (*n* = 2), precision farming (*n* = 2) and renewable energy production. Study authors reported carbon footprint-reducing practices to positively impact biodiversity in 65% of studies, to have mixed effects in 11%, negative in 8% and no effect in 16% of studies. As no critical appraisal was carried out on the included studies, we recommend further study validation and synthesis in order to support these findings.

**Conclusions:**

The evidence base has highlighted evidence clusters and gaps on how farming practices that can reduce the carbon footprint of a farm impacts agriculturally associated biodiversity. There are many areas for further research including studies investigating the long-term relationship of interventions that alter habitats over a long period such as rewetting peat soils and increasing tree cover. Future research should observe abundance and diversity of multiple species to generate a better understanding of an intervention’s impact. The review evidence base largely matched the primary evidence base, however none were conducted with systematic methodologies. This systematic map is intended to direct further primary and secondary research to improve the understanding of how carbon footprint reducing practices impact biodiversity, thus contributing towards meeting the legally binding global environmental targets in concert.

**Supplementary Information:**

The online version contains supplementary material available at 10.1186/s13750-025-00372-7.

## Background

Changes to land management are critical and time-bound in meeting the commitments to the Convention on Climate Change and the Convention on Biological Diversity [1]. For instance, the UK must be carbon net zero (CNZ) by 2050 [2] and have reversed the decline in nature by 2040 [3]. In many countries with high population density, land is a premium commodity, the possession of which is in demand for many sectors. Agriculture is often the dominant land use [4] which is under pressure to increase efficiency to feed rising populations and keep food costs low [5]. It is unlikely that large increases in land will be made available for food production given concurrent demands for increased forestry, housing development, recreation, conservation and other uses such as renewable energy production. Existing agricultural land must therefore balance the requirement to produce more food with contributing to environmental targets. Currently however, agriculture appears to be detrimental to, rather than in support of, CNZ and nature recovery [5].

Indicator species monitoring, used as a proxy to indicate overall biodiversity within an ecosystem [6], reveals that European farmland biodiversity has been on a downwards trajectory for decades. In England, for example, the abundance of farmland birds has declined by 61% from 1970 to 2022 [7]. Largely to blame is market force-driven intensification to maximise the productivity of land and resources [8]. Post World War II intensification resulted in widespread losses of seminatural habitats that was accelerated by technological advances [5, 9, 10]. Agricultural field sizes have increased due to the removal of hedges, and previously unfarmed areas have been brought into cultivation by the capabilities of increasingly sophisticated farm machinery [11]. Seminatural habitats on farms, such as hedges, provide food shelter and connectivity for wildlife to thrive. Many species aid agricultural production through the provision of ecosystem services, such as the pollination of crops [12], pest control [13, 14] and soil health [15]. Declining numbers of these species have reduced these ecosystem services [16] and have fuelled an increase in the application of pesticide agrochemicals (herbicides, fungicides and insecticides). Many pesticides have been reported to harm nontarget species [17, 18], further negatively impacting on-farm biodiversity.

Market forces have also influenced what farmers grow. Many farms grow a reduced variety of predominantly high-value crops, when compared with pre-supermarket times, when local farms supplied a range of products to local markets [9, 10, 19]. A reduction in the number of crops grown, in a landscape, can reduce habitat provision and functional diversity. This effect is evident in the 90% decline of traditional orchards since the 1950s which once provided nesting sites for tree dwelling species and a provider of nectar for pollinators at different times of year on farms [20, 21].

In addition to contributing to the decline in biodiversity, agriculture accounts for 9.4% of United States of America’s and 11% of total European Union and UK greenhouse gas (GHG) emissions [22–24] despite contributing a relatively small amount directly to gross domestic product (GDP) (0.56% UK GDP [25]), although indirectly the agri-food sector contributes 6.5% to UK Gross Value Added. The majority of GHG emissions in temperate agricultural production systems are methane from ruminant cattle, nitrous oxide from artificial fertilisers and carbon dioxide from farm machinery and soils [23, 26, 27]. The UK’s Climate Change Committee (CCC) reports that if the UK is to meet its 6th carbon budget, the agricultural sector must reduce emissions by 10 MtCO_2_e (approximately a 20% reduction from a 2019 baseline by 2033) whilst recognising that agriculture will be a sector where residual emissions will need to be offset by carbon removals because of unavoidable biogenic emissions [28]. Agricultural emissions have stagnated over the previous decade which could indicate that current policies to reduce the carbon footprint of farming are having little effect [27]. However, productivity has increased in many agricultural sectors. This is evident in the dairy sector. Production has increased by 13% since 1990, whilst the number of livestock has reduced by 21% [22]. Therefore, the carbon footprint of a litre of milk is lower, but overall dairy sector emissions have not significantly reduced.

The actors in farming’s transition to net zero carbon emissions includes governments, industries who include agricultural emissions as part of their Scope 3 (GHG) emission reporting [29] and farm support groups. There is large consensus between the different stakeholders on the methods proposed to achieve this shared aim. In 2019, the National Farmers Union (NFU) [30] published a report on how farmers can reach CNZ. Land management objectives to reach CNZ have been included in the Institute for European Environmental Policy and UK’s CCC most recent policy suggestions for land use [31, 32].

Many of the land management objectives being promoted involve land use change or land management change. Therefore, they have the potential to alter habitat provisions (such as food and shelter) within agroecosystems, thus impacting biodiversity. For example, increasing tree cover on agricultural land, such as through agroforestry, is being promoted to increase overall carbon sequestration/removal (contributing to CNZ [32]) and create new habitat [3]. Similarly, other promoted interventions have the potential to change habitats while reducing the carbon footprint of agriculture. This includes actions to improve soil health, such as avoiding soil compaction, precision farming, increasing soil organic carbon (SOC) and reducing synthetic fertiliser application [33]. Growing biomass for bioenergy production and the generation of renewable energy will also alter agricultural landscapes [34]. Although, to comply with Article 1b of the Paris Agreement (to limit global warming but not at the expense of food production [35]), focus should be on converting less productive areas for this purpose. Similarly, the restoration of drained, highly fertile/productive agricultural peat soils involves raising the water table to reduce GHG emissions [36]. Thus, resulting in considerable habitat changes to the agroecosystem. The CCC [31] suggested that 50% of upland peat and 25% of lowland peat should be restored. This includes investigating the viability of continuing to farm rewetted peat soils for food and fibre, known as paludiculture.

Stakeholders that influence agricultural production need to understand the full impact of the pressures that are being imposed upon agriculture. Intensification of agricultural land in the mid-twentieth century instigated a change in the landscape without full knowledge of its potential for environmental damage [19]. Therefore, a drive towards a carbon or a nature recovery target al.one, should be cautious of wider sustainability implications.

### Theory of change

Many of the interventions that are being encouraged to reduce the carbon footprint of agriculture, such as growing crops for bioenergy, will impact farmland species through changes to habitat provision and habitat management [33, 34, 36]. Understanding this relationship is imperative to ensure that CNZ and nature recovery targets are met. For example, agroforestry and other interventions to increase tree cover on farmland are widely promoted as tools to reach CNZ [31]. Trees are beneficial to many species and can be considered good for biodiversity [37]. However, tree establishment can be detrimental to on-farm biodiversity, as it requires protection from mammal browsing and protection from competing weeds, often requiring chemical control. Therefore, an understanding of a tree’s lifetime impacts on biodiversity is needed. Additionally, understanding how increasing tree cover impacts all species within and surrounding agricultural land is needed. For example, trees and hedges provide an ideal habitat and ecotone for many birds, but in areas highlighted for ground-nesting species conservation, such as lapwing (*Vanellus* spp.), this can have a negative effect, as increasing tree cover results in their displacement due to increased predation [38].

Our understanding is that at this point, no systematic map has been undertaken to gather all relevant evidence on how widely adoptable carbon footprint-reducing interventions impact biodiversity on temperate agricultural land. A comprehensive, transparent, objective evidence synthesis of this subject is needed to inform stakeholders of agricultural land to make informed decisions about how to reverse the decline in biodiversity whilst reaching CNZ. To understand an intervention’s full impact on biodiversity, assessments of its impact on multiple species, including those of principle importance need to be performed. Therefore, a systematic map is proposed to understand what evidence exists on how carbon footprint-reducing interventions impact organisms on agricultural land and surrounding agroecosystems. The findings of this map are intended to be used by agricultural land stakeholders, including farmers and policy makers, to implement or promote interventions to meet local environmental goals to contribute to global environmental targets.

### Stakeholder engagement

The systematic map title and research question were presented at a stakeholder meeting (see ‘Funding’ section) where the stakeholder group discussed the purpose and importance of the research in the context of a wider research project.

## Objective of the review

The objective of this review was to identify and systematically map research investigating how land management objectives that are promoted to lower GHG emissions or increase carbon sequestration impact biodiversity on agricultural land and associated agroecosystems. The map is restricted in geographic scope to temperate climates and farming systems like those of the UK (e.g. grassland grazing, arable, horticultural and viticulture).

### Primary question

Primary Question: What evidence exists that the adoption of agricultural practices to reduce a farm’s carbon footprint impacts on-farm biodiversity?

## Methods

This systematic map followed the Collaboration for Environmental Evidence (CEE) guidelines [39] and complied with the Reporting Standards for Evidence Synthesis (ROSES) [40] (Additional File 1). The protocol for this map was published on PROCEED [41]; some deviations from the published protocol were required, these are discussed in the next section.

### Deviations from the protocol

#### Searching

The Scopus database was included to increase the comprehensiveness of the search. The first 20,000 articles sorted by relevance were exported, as that was the limit of the database.

#### Screening

The inclusion criteria for climate were restricted to temperate areas of Europe, North America and New Zealand. Owing to the volume of articles, a double screening (where the same study is assessed by two reviewers to check the precision of the inclusion criteria) of the title and abstract was carried out on 2% (850) of articles rather than 5% as stated in the protocol.

#### Data coding

Minor changes to the coding strategy were made to streamline the coding process. The longitude and latitude of a study location were not recorded; rather, the country of the study was recorded. Coding of the author reported outcome of the study was changed from ‘yes/no’ to report if the effect that was reported by the authors were positive, negative, mixed effect (where some species benefit, and others are disadvantaged by an intervention) or there was no significant change to the abundance and/or diversity of the studied population (shown by ‘+’ or ‘−’ or ‘+/−’ or ‘~’ respectively in Additional File 4).

### Search for articles

Three publication databases were identified as suitable for this research question during a meeting of the authors: WoS, Scopus, and EBSCOhost (which includes CAB Abstracts).

Searches for peer reviewed articles and grey literature were carried out via a search string that was developed through a scoping process; the details of how the final search string was developed can be found in Additional File 2 of the protocol [41]. The search terms were identified by analysing the components of the PICO (see Eligibility Criteria), from benchmarking articles and discussions within the stakeholder group. The search terms for carbon footprint-reducing interventions were developed from promoted interventions within the UK CCC Land Use Policy Framework [31], the majority of which are cited as being identified in the NFU publication ‘Achieving Net Zero Farming’s 2040 goal’ [30]. The synonyms and interpretation of generic interventions to be included in the search string were discussed in a meeting of the authors. All searches were conducted in English. No date restrictions were applied to the searches. The exact details of each search can be found in Additional File 2.

#### Internet searches

As stated in the protocol, we investigated the use of Google Scholar to identify relevant literature that may not be found by the search string. However, the search string was too large for the algorithm to work. Web-based search engines often change their algorithms and learn from previous searches, which reduces transparency and replicability [42]. Google scholar was not included in the searches.

#### Specialist sources

A meeting of the authors generated a list of organisation websites with potentially relevant articles, including the Agricultural and Horticultural Development Board, the British Ecological Society, the Chartered Institute of Ecology and Environmental Management, Gov.uk, the Game and Wildlife Conservation Trust, the Royal Society for the Protection of Birds, the UK Centre for Ecology and Hydrology, the US Department for Agriculture, and the Community Research and Development Information Service. Searches on the websites using keywords were carried out on the 11th and 12th of April 2024. References of articles that met the inclusion criteria were recorded and added to EPPI-Reviewer-Web. Details of the search strategy can be found in Additional File 2.

#### Supplementary searches

No supplementary searches were undertaken.

#### Comprehensiveness of the search

The comprehensiveness of the search was determined by testing whether ten known relevant articles (listed in Additional File 3 of the protocol) would be found by the search string. When articles were missing, the search string was altered to include relevant search terms to capture the benchmarking articles. The search string identified all ten articles in the WoS database. The comprehensiveness of the search was improved by the inclusion of a trawl of relevant websites for grey literature (defined as research or information that are not peer-reviewed articles).

#### Assembling and managing search results

The results of the database searches were downloaded as RIS files and imported into EPPI-Reviewer-Web. Once all the references were collated, duplicates that had a similarity score of 0.85 or greater were removed via the automatic function. Articles with similarity scores of 0.7–0.85 were resolved by manually assessing them to avoid false duplicates.

### Article screening and study eligibility criteria

#### Screening process

**Fig. 1:**
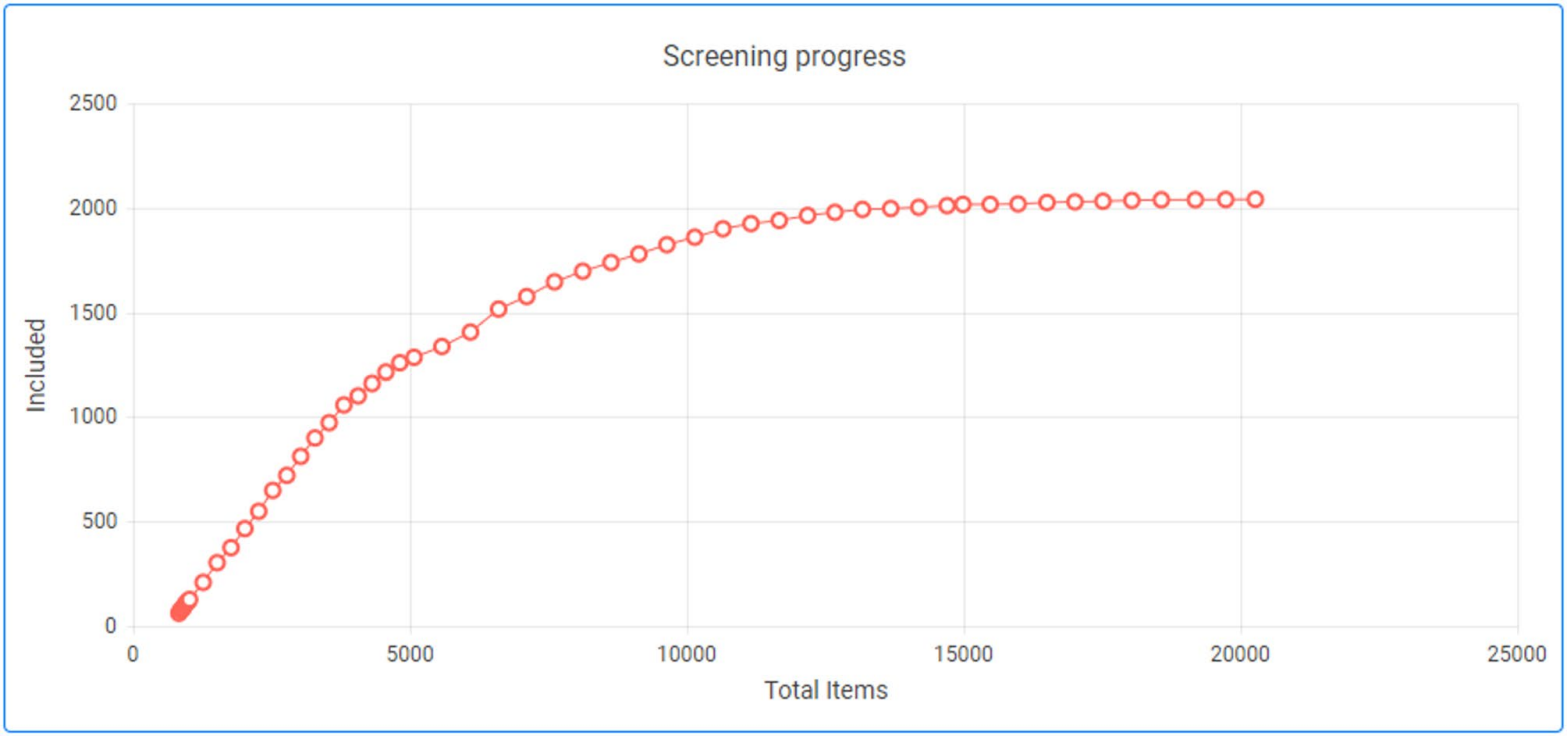
Inclusion progress of screening at title and abstract using the priority screening function on EPPI-Reviewer-Web. The inclusion progress of the first 850 articles is not represented as priority screening was not enabled. Red circles indicate the start and finish of a screening session, the red line indicates inclusion progress

Following the removal of duplicates, two reviewers screened the first 850 randomly selected articles at the title and abstract level to assess the replicability of the eligibility criteria. Reviewers’ consistency was assessed via Cohen’s kappa test [43], to indicate the level of agreement between reviewers. The inclusion/exclusion decisions were the same for 98% of the double-screened articles, which resulted in a Cohen’s kappa of 0.82, which was interpreted as near perfect agreement. Neither reviewer has published material, so there was no chance of screening their own work. CEE guidance [39] states that all articles should be screened by two or more reviewers, however, owing to the resources available for the evidence map, the remainder of the articles were screened by one reviewer. The priority screening function on EPPI-Reviewer-Web was employed to assist title and abstract screening. This is an active learning-powered priority screening which works by learning the differences between those articles that are included or excluded. Priority screening then reveals articles that are likely to be included. This function was used after consistency checking when there was a large pool of decisions to learn from (850 screened articles (Fig. 1). The decision to stop screening was made when the inclusion percentage dropped below 1% which occurred after 20,312 articles were screened and 2040 articles were included for full-text screening. Full-text screening was then carried out. The reason for an article’s exclusion at this stage was recorded (Additional File 3).

#### Eligibility criteria

At each stage of screening, the articles were assessed for eligibility against the inclusion criteria detailed below. If there was no reason or an insufficient reason to exclude an article, it was passed to the next level of screening until ultimately being included in the map.

Population: A study addresses organisms on agricultural land and surrounding agroecosystems, e.g., adjacent watercourses. Only temperate climate regions within Europe, North America and New Zealand were included. Articles that described the climate as Mediterranean, arid/semiarid, subtropical or boreal were excluded.

Interventions: Adoption of practices on temperate agricultural land that reduce GHG emissions or increase carbon sequestration/removal as promoted by the UK CCC Land Use Policy Framework. Practices to: avoid compacted soils, produce bioenergy, enhance hedgerows, increase SOC stocks, increase tree cover, restore peat soils, adopt precision farming, reduce fertiliser input, generate renewable energy were included. For example, controlled traffic farming, cover cropping and silvoarable systems are interventions that were eligible interventions (all practices are listed in Fig. 7). An eligible intervention must involve land use change or land management change and must be conducted within similar farming systems to those that are commercially adopted in the UK.

Comparators:

- Before adoption of low-carbon farming practices or increased sequestration/removal.
- A control site where the adoption of low-carbon farming practices or increased sequestration/removal have not been applied,
- A comparison between low-carbon farming practices or increased sequestration/removal (if a suitable control exists in this instance, the interventions will be recorded as different studies).
- A comparison of differing levels of a practice, such as different types or mixtures of cover crops.
- A time series at the same site after adoption of low-carbon farming practices or increased sequestration/removal.

Outcomes: Measured or observed changes in organism diversity and/or abundance.

Eligible types of study design: Only primary studies and review articles that involved field-based manipulations or comparative observations were included. Only studies that have quantified biodiversity changes on agricultural land and associated agroecosystems were included. If there was a comparison between interventions or different levels of an intervention, this was coded as one study. Where studies were replicated across multiple geographic locations, they were coded as one study. Some articles contained multiple studies. Reviews and meta-analyses were recorded in a separate database.

Any additional criteria: Only English language studies were included. All articles where the population is studied within farming systems not commercially viable in the UK, e.g., cotton (*Gossypium* spp.), were excluded.

#### Study validity assessment

The study publication type, design and duration of primary studies were recorded and collated to indicate the validity of the evidence base. The review articles in the evidence base were coded according to review type (e.g. narrative review and meta-analysis). If a review was listed within the Collaboration for Environmental Evidence Database for Evidence Reviews (CEEDER) [44] at the time of coding this was recorded. The risk of bias in the methods of these reviews were assessed by collating the scores it received from a Collaboration for Environmental Evidence Synthesis Assessment Tool (CEESAT) assessment [44]. CEESAT assigns a score, awarded by two reviewers, for 16 features of each evidence review to indicate the reliability of the evidence synthesis. Gold is the highest possible score, followed by green, then amber and red as the lowest.

#### Data coding strategy

Studies were coded in Microsoft Excel using a drop-down menu of inputs for each metadata (Additional File 4). A study was determined by the presence of one population group and one intervention. Some articles contained multiple studies. Double coding (where two authors coded the same articles to check the replicability of the coding strategy) was carried out on the full texts of 5% of the articles included at full text screening. Initially, a subset of five articles were double coded and before the remaining articles of the subset were coded, the minor discrepancies were discussed and resolved. Overall, double coding provided a high similarity (95%) between coders.

#### Data mapping method

The database of coded primary research and reviews (Additional File 4) was mapped through descriptive analysis using tables, graphs and charts. They describe the bibliographic and methodological metadata of the studies. Graphs charts and heatmaps were also used to describe the elements of the study that are relevant to the PICO of this evidence base. MapChart [45] ​was used to show the distribution of the study locations by country.

## Review findings

### Review descriptive statistics

**Fig. 2:**
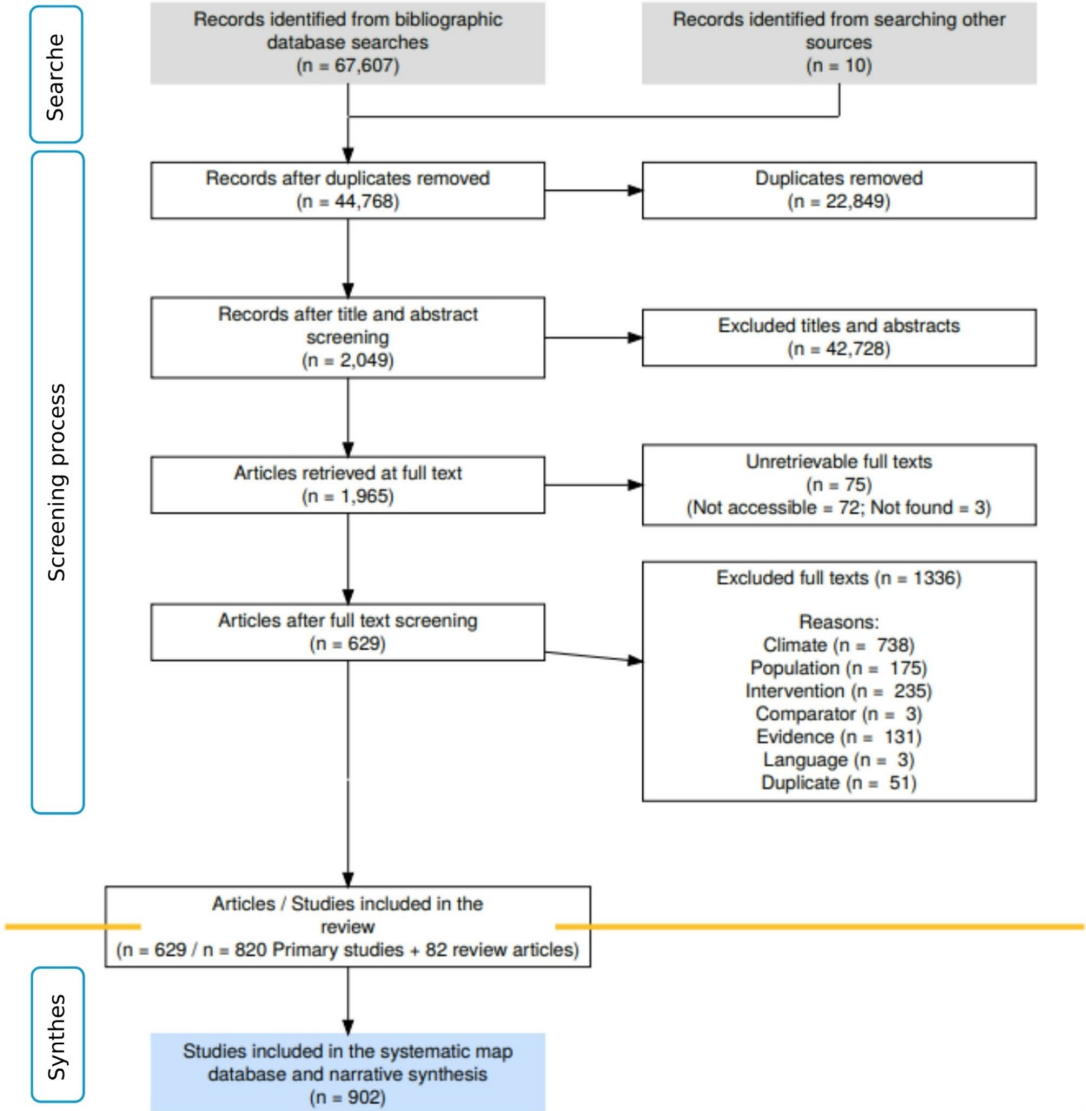
ROSES flow diagram showing the number of articles at each stage of the evidence mapping process

In total, 67,617 articles were found during the search for evidence that took place between 08/04/2024 and 12/04/2024. The first 20,000 articles sorted by relevance were extracted from Scopus, 36,600 from WoS, 11,007 from CAB abstracts, and 10 articles were found on organisation websites. A ROSES flow diagram (Fig. 2) describes how the evidence base was created. Once duplicates were removed 44,768 articles remained. A total of 20,312 titles and abstracts were screened. The screening of the full texts revealed 547 eligible articles to be included in the evidence base. The main reasons for exclusion from the full-text screening were climate (52%) and intervention (17%). The reasons for the exclusion of articles from the full-text screening are shown in Additional File 3. Some articles contained more than one study, resulting in a total of 820 studies being coded.

### Characteristics of the evidence base

#### Publication type

Of the included studies 93% (*n* = 759) were peer-reviewed, 7% (*n* = 58) were conference reports, and 0.4% (*n* = 3) were project summaries.

#### Geographic location

**Fig. 3:**
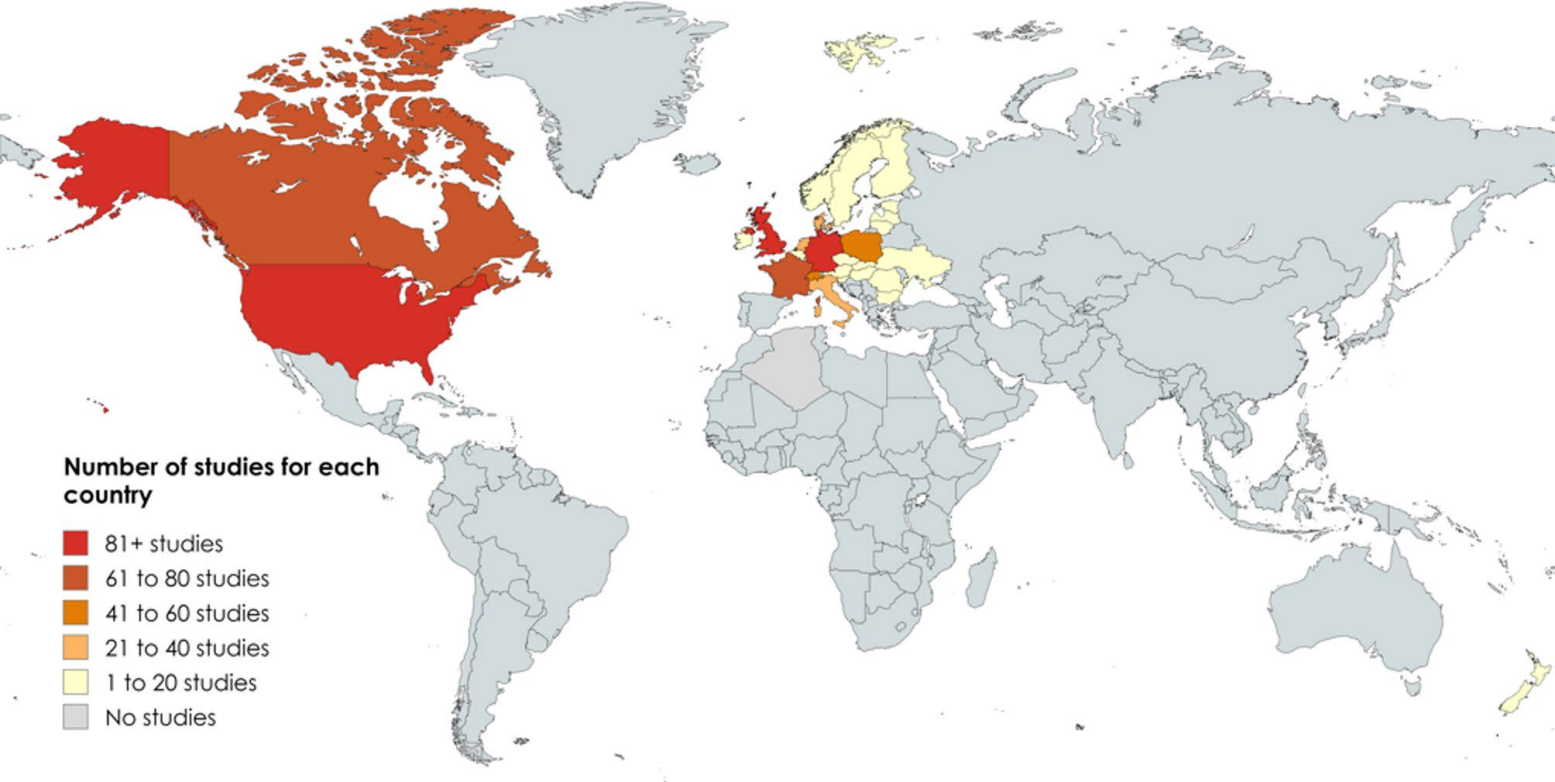
Number of included studies by country. In descending order, UK = 150, Germany = 118, USA = 82, Canada = 78, France = 73, Switzerland = 48, Poland = 46, Italy = 33, Denmark = 21, Holland = 20, Austria = 19, Czech Republic = 18, New Zealand = 17, Hungary = 16, Ireland = 15, Lithuania = 15, Romania = 15, Belgium = 14, Sweden = 12, Estonia = 10, Slovenia = 7, Latvia = 6, Norway = 5, Finland = 4, Slovakia = 3, Bulgaria = 1, Ukraine = 1, and all other countries = 0 included studies (Source: MapChart.com [45])

The inclusion criteria restricted studies to those of a temperate climate and farming production system, similar to that of the UK. The UK contributed 150 studies (18%), the most from an individual country. The second largest contributor was Germany, with 118 studies (14%), followed by the USA, Canada and France, with 82, 78 and 73 studies, respectively. These five locations (highlighted in red and dark orange in Fig. 3) contributed 59% of the evidence base (*n* = 501). A total of 847 locations were recorded, as some studies were replicated in different geographic locations and were coded as one study. Not all areas of a country met the inclusion criteria and did not contribute to the evidence base such as semi-arid areas of North America.

#### Publication year

**Fig. 4:**
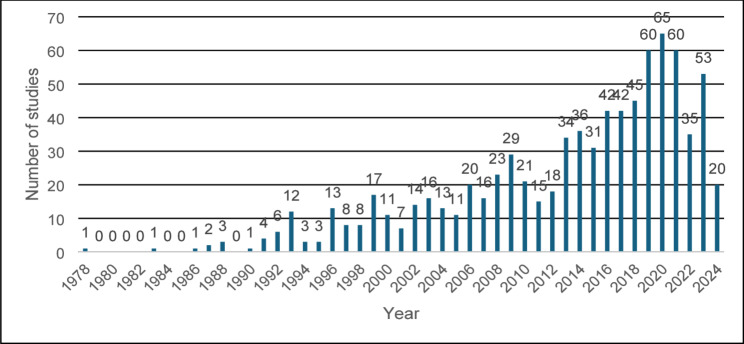
Number of studies by publication year. The representation for 2024 is not for the whole year as searches for evidence will only have included articles published before April

The first included study was published in 1978 [46]. Figure 4 shows how the number of published studies per year increased from 1978 to 2022 and peaked in 2020. The years after 2020 did not follow the general trend of increasing numbers of studies and were potentially impacted by restrictions to experimental work resulting from COVID-19. The searches for this evidence database were carried out in April 2024. Therefore, this map does not include studies published beyond that date, and the 2024 bar should not be considered representative for the whole year.

### Mapping the quantity of studies relevant to the question

#### Agricultural system

**Fig. 5:**
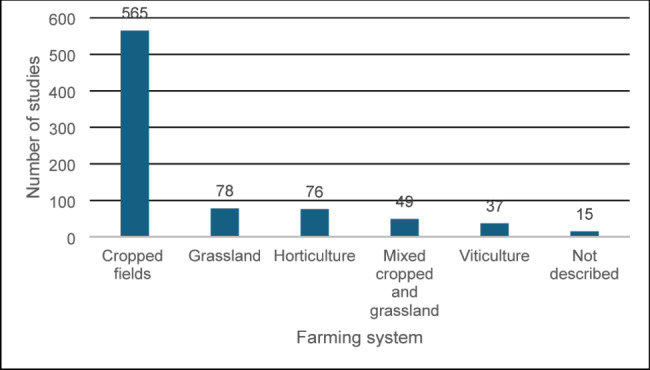
Number of studies by farming system

Studies carried out on arable cropping systems were the most numerous (*n* = 565), followed by grassland studies (*n* = 78) and horticulture studies (*n* = 76) (Fig. 5).

#### Population

**Fig. 6:**
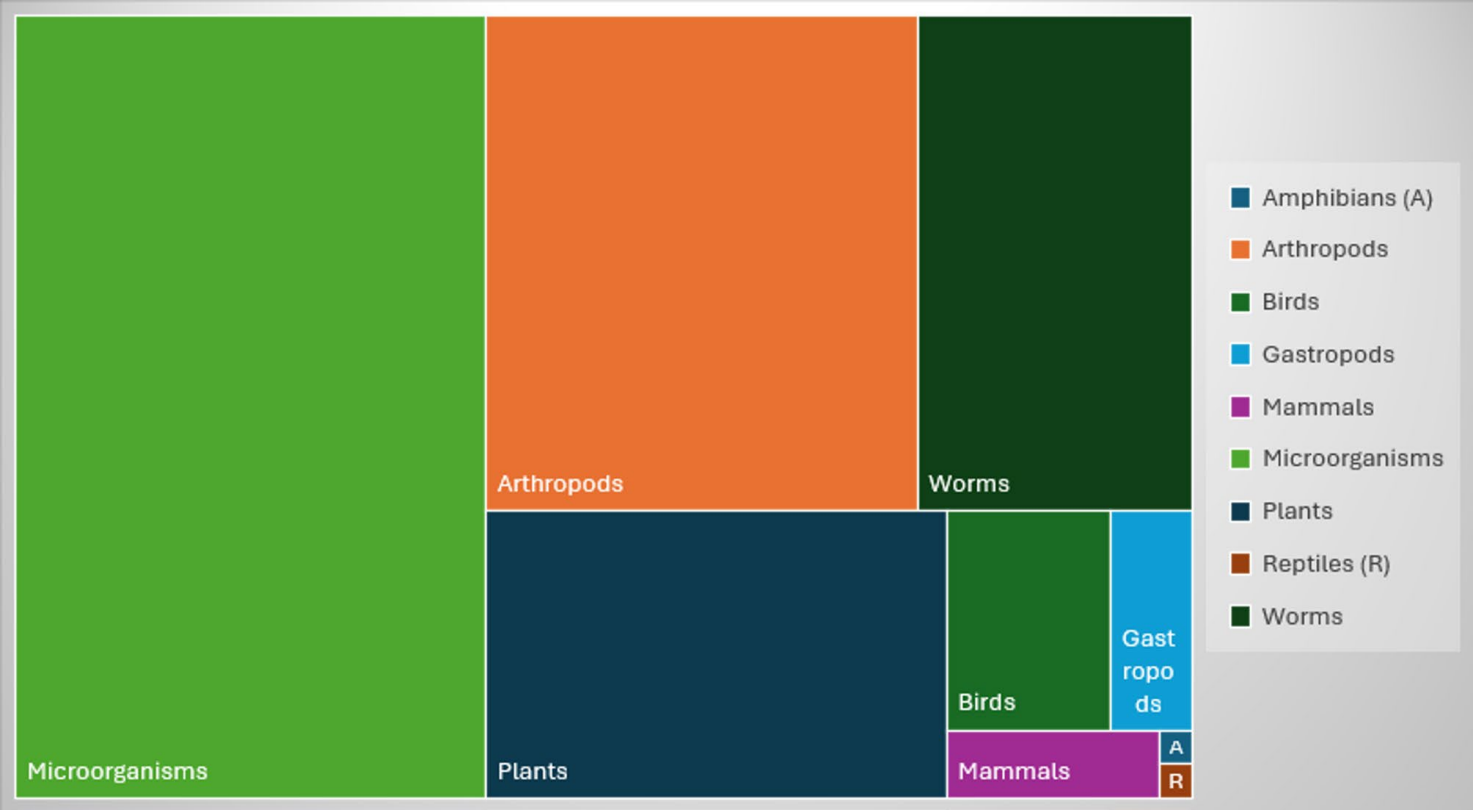
Tree map of the relative proportion of observed taxonomic population groups in the evidence base. Microorganisms (*n* = 328), arthropods (*n* = 190), worms (*n* = 120), plants (*n* = 118), birds (*n* = 32), gastropods (*n* = 17), mammals (*n* = 13), amphibians (*n* = 1) and reptiles (*n* = 1)

The population was coded by taxonomic group. Figure 6 displays each group, showing the proportionate size of the number of observed studies. Further information on the species family or name was recorded in the coding sheet if stated in the study (Additional File 4). Microorganisms were included the most in the evidence base as they were observed in 40% of included studies. Arthropods were the second most common, with a 23% proportion, followed by worms and plants, which accounted for 15% and 14%, respectively. Studies in which birds were observed accounted for 4% of the evidence base. Gastropods (2%) and mammals (1.6%) were observed in relatively few studies. Amphibians and reptiles added only one study each to the evidence base (0.1% each shown at the bottom right of Fig. 6).

#### Interventions to meet land management objectives

**Fig. 7:**
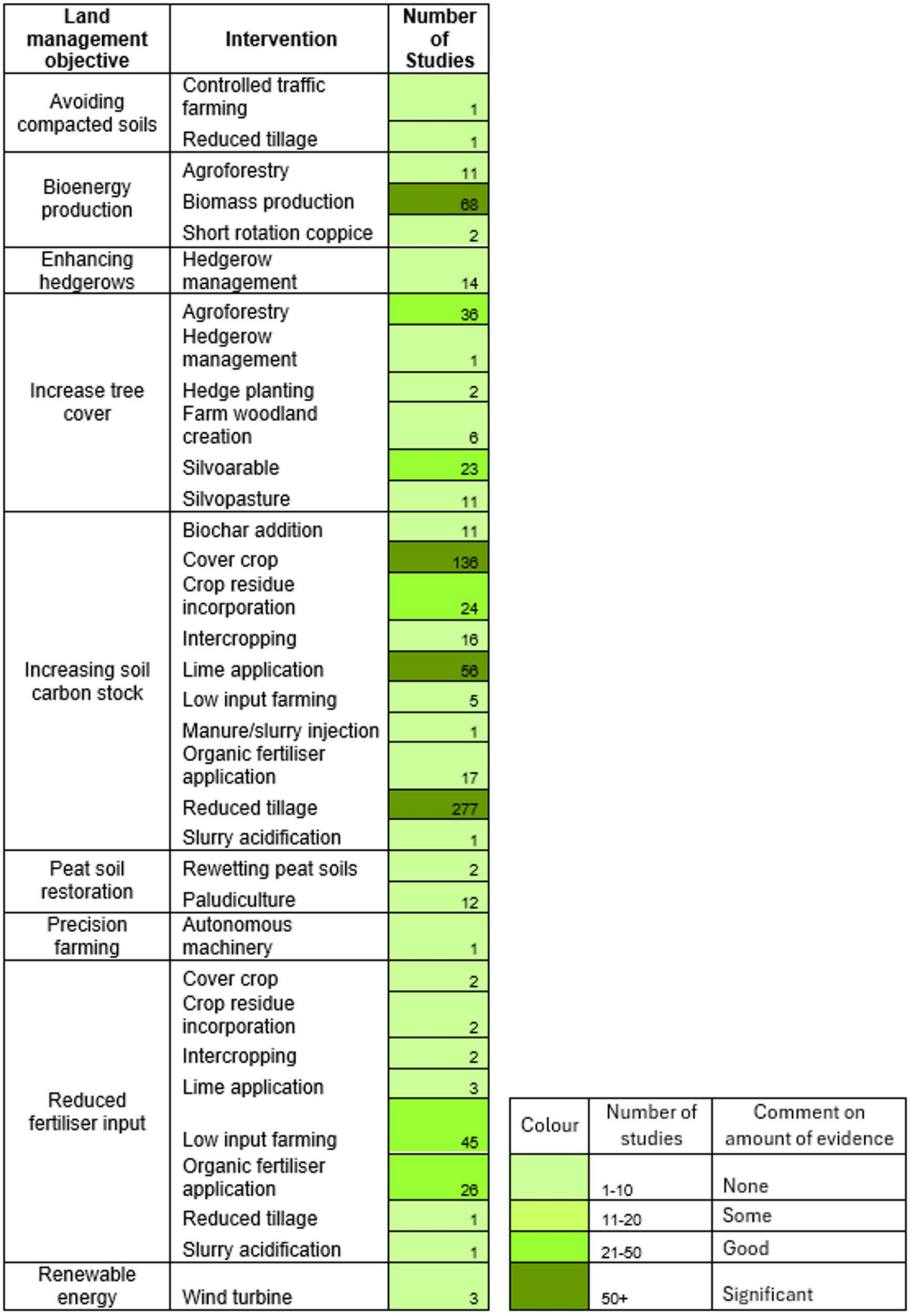
The number of studies per intervention for each land management objective. Darker shading indicates a larger body of evidence. Some interventions appear more than once, as they fall into multiple categories

Land management actions were coded and then subcategorised into interventions that met the purpose of the land management action. An indication of the amount of evidence related to an intervention is denoted by colour coding in Fig. 7. Most studies (*n* = 544 or 66%) have investigated increasing SOC impacts biodiversity. Reducing the amount of fertiliser (*n* = 82), increasing tree coverage on farms (*n* = 81) and producing bioenergy material (*n* = 79) each contributed approximately 10% to the evidence base. Interventions to enhance hedgerows for carbon sequestration/removal and restoring peat soils were included the same number of times (*n* = 14). Both added 1.7% to the evidence base. Few studies related to energy production (*n* = 3, excluding bioenergy material production), avoiding compacting soils (*n* = 2) and precision farming practices (*n* = 1) were included. No agrivoltaic (e.g. grazing within solar farms) studies were found. Agroforestry captured all interventions related to planting trees on agricultural land not already described (e.g. shelterbelts, field trees and riparian buffers). Some interventions feature in multiple intervention groups. For example, the adoption of agroforestry increases tree cover on farms but can also provide biomass for bioenergy production.

### Mapping the quality of studies relevant to the question

#### Study design

**Fig. 8:**
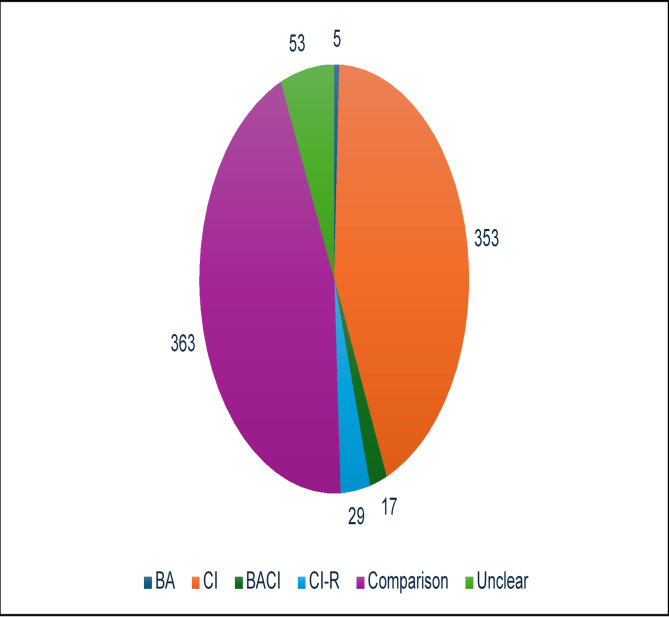
Number of studies by study design. *BA* Before–After, *CI* Control-Intervention, *BACI* Before–After-Control-Intervention, *CI-R* Randomised Control

The studies were coded according to the experimental design (Fig. 8). Studies with a control made up 49% of the evidence base, although some studies may have contained one or more versions of the same intervention. Of those studies with a control, five used temporal control (before-after), 353 studies had spatial control (control-intervention), 17 studies employed both spatial and temporal control (before-after-control-intervention), and 29 studies utilised a randomised control. Some studies had a comparator (e.g. different levels or types of intervention) but contained no control. These accounted for 363 studies in the evidence base (44%). Most studies with an unclear study design were those coded from their abstract only (*n* = 51) and two conference reports that were included had unclear study designs.

#### Study length

**Fig. 9:**
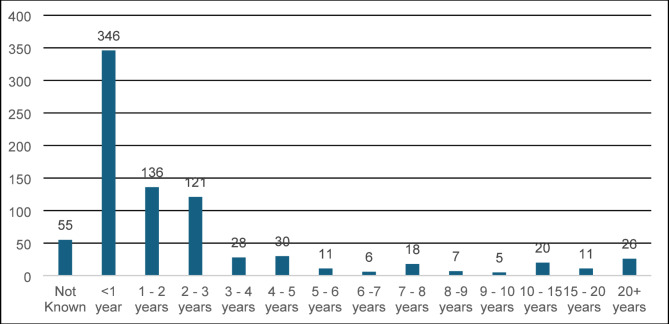
Number of studies by length of data collection

The length of time the data were collected for each study was recorded (not how long the intervention was in place). Figure 9 shows that the most common study length was less than 1 year (*n* = 346). Most studies, 81%, had a duration of less than 5 years. Long-term studies, which were over 8 years in duration, accounted for 8% of the evidence base.

#### Study length for each intervention

**Fig. 10:**
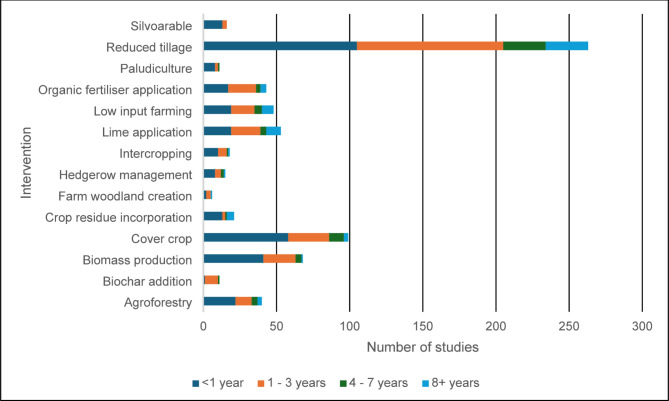
The number of studies per study duration for interventions represented more than five times in the evidence base

The length of time each intervention was studied in the literature ranged from less than 1 year to over 20 years. Figure 10 shows this variation in length of study for any intervention that appeared more than five times in the evidence base. Reduced tillage appears to provide the most robust evidence base, as it is the most studied intervention and has a range of study durations, of which over 60% were for more than 1 year. Cover crops, crop residue incorporation, lime application and low-input farming interventions also provide a potentially robust evidence database because of the mixture of study durations, and approximately 40% of the studies were longer than 3 years.

Agroforestry and biomass production are well represented in the evidence base (Fig. 7). However, 75% of the agroforestry studies had a duration of less than 3 years, and only three studies had durations longer than 8 years. Other interventions to increase tree cover on farms do not include a study length of over 3 years (hedgerow planting, short rotation coppice, silvoarable and silvopasture). The impact of biomass production on biodiversity was reported for less than 3 years in more than 90% of studies, and one study lasted longer than 8 years.

### Knowledge gaps and clusters

#### Land management objective and population groups

**Fig. 11:**
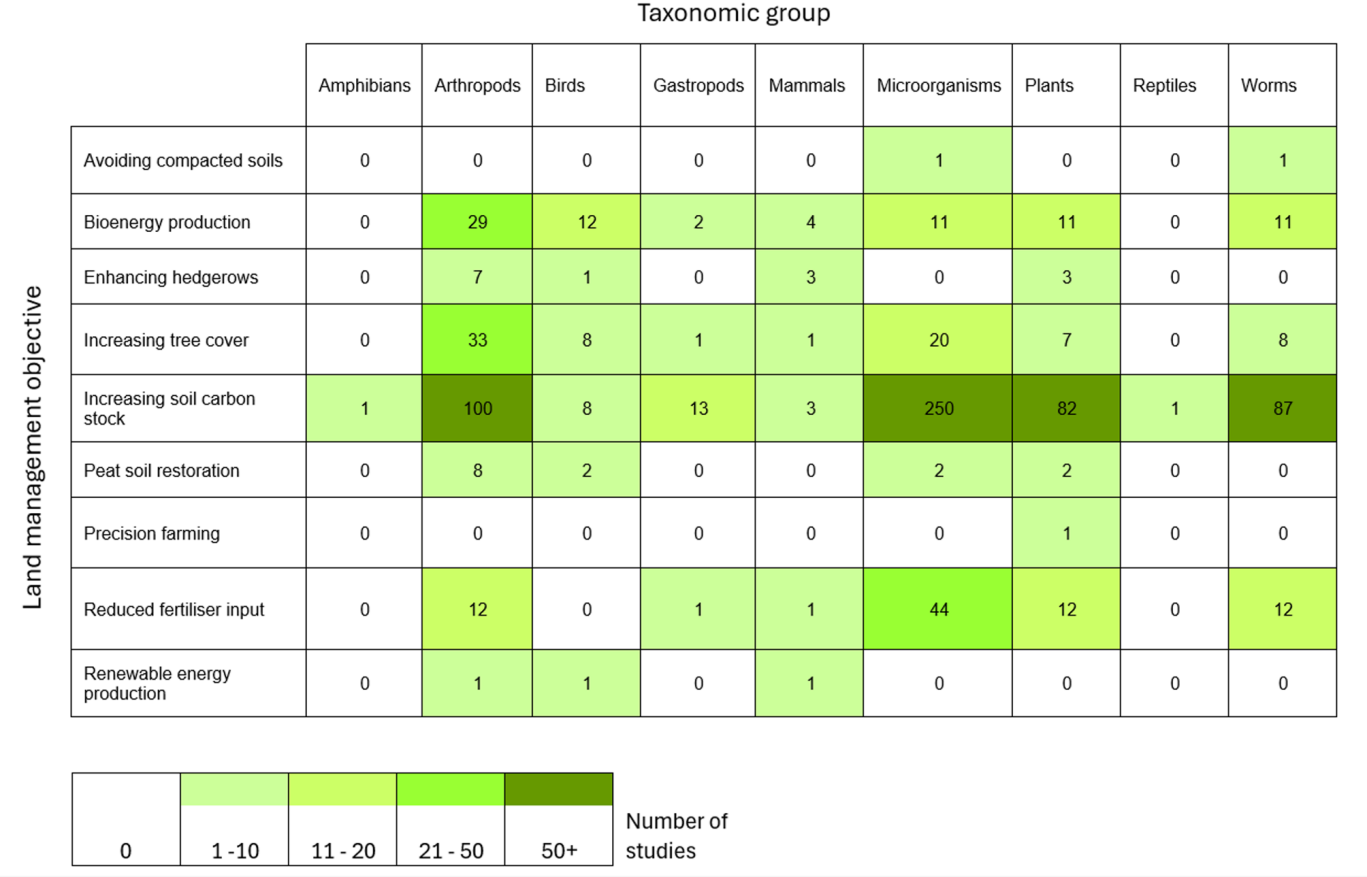
Heatmap of studies carried out by land management objective and population group

Recording the number of studies that investigated interventions to achieve carbon footprint reducing land management objectives against a taxonomic group produces clear knowledge clusters (highlighted in dark green in Fig. 11) and knowledge gaps (white and pale green boxes in Fig. 11). The majority of the evidence base (63%) is made up of investigations on how increasing SOC impacts microorganisms (*n* = 250), arthropods (*n* = 100), worms (*n* = 87) and plants (*n* = 82).

#### Author reported outcomes

**Fig. 12:**
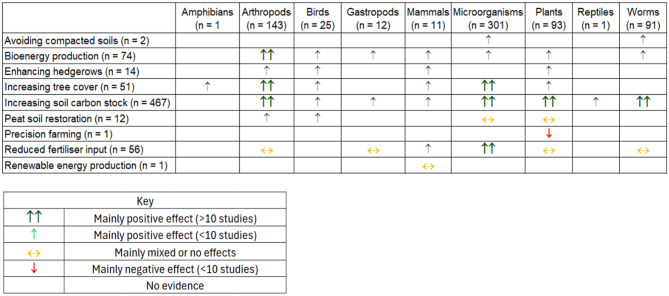
Authors reported impacts on different taxonomic groups from studies of land management actions to reduce the carbon footprint of farming. The effect relates to a change in the abundance and/or diversity of the measured species i.e. a positive effect means an increase, negative effect means a decrease

Figure 12 shows a synthesised score of authors reported outcomes on how carbon footprint-reducing land management objectives impact the abundance and/or diversity of taxonomic groups. Only studies available at full text were included in this analysis. Most land management objectives to reduce the carbon footprint of agriculture indicate a positive interaction with biodiversity, although peat soil restoration, reducing fertiliser inputs and renewable energy production were more likely to be reported as having mixed effects. This vote counting exercise should be built upon with quality appraisal and further synthesis (for example meta-analyses of specific intervention/outcome relationships) to improve the confidence in these findings, and to establish the accuracy of the effect directions and calculate the size of any effects. Most studies investigating microorganism populations reported a change in the abundance and diversity of populations relating to an intervention (81%). However, there were 57 studies (19%) that describe a change in the composition of the population of microorganisms (coded as ‘~’ in Additional File 4). Although land management practices to avoid compacted soils, produce bioenergy, enhance hedgerows, increase tree cover and increase SOC generally indicate a positive impact on biodiversity, the increasing populations of some species may not be considered beneficial to agricultural productivity (e.g., increasing populations of pest species such as gastropods). For example, of the studies investigating plants, 85% were studies observing an intervention’s impact on weed populations, which would be considered a negative impact by many farmers. In contrast, the study investigating precision farming techniques [47] reported a reduced abundance of weeds (a negative outcome for plant abundance and diversity) which was reported in a positive manner by the authors. A similar effect was reported in 14 studies investigating cover cropping.

**Fig. 13:**
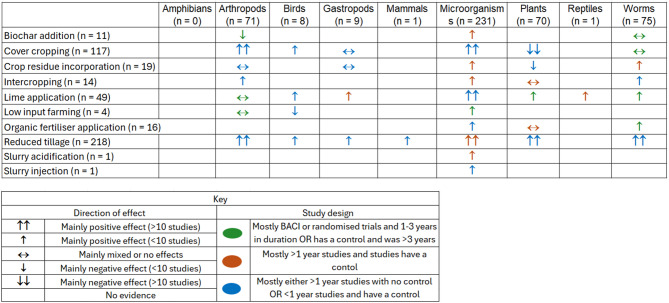
Synthesised authors reported outcomes from the evidence of how interventions to increase SOC impacts taxonomic groups. The effect relates to a change in the abundance and/or diversity of the measured species i.e. a positive effect means an increase, negative effect means a decrease. The study design and duration were also synthesised for each cell to indicate the reliability of the evidence indicated by the colour of the symbol

The most commonly studied interventions were those that aim to increase SOC. The author-reported outcomes for these are summarised in Fig. 13.

For the SOC studies, a limited quality appraisal was carried out based on length of study and presence/type of control. Reduced tillage was intervention most consistently reported (across taxonomic groups) to have a positive impact on diversity/abundance, although studies were less likely to be long-term or have a control than some other interventions. The findings were, however supported by meta-analyses that were also included in the map. Of the 14 meta-analyses that synthesised evidence on how interventions to increase SOC stocks impacts biodiversity, nine meta-analyses found reduced tillage to be benefical to biodiversity. Meta-analyses that synthesised cover cropping evidence reported either positive outcomes for biodiversity (*n* = 4) or mixed effects (*n* = 1), although none of these investigated plants as a population.

The outcomes from studies investigating reduced tillage impacts on microorganisms, appears to be the most robust evidence base, as 68% of the large evidence base (*n* = 79) studies were multiyear studies and had a control (rather than a comparative study).

### Reviews

**Fig. 14:**
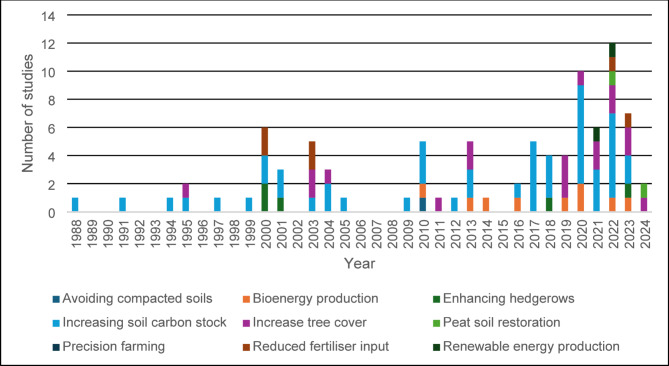
Number of reviews by publication year

During the screening process, 82 review articles were identified and coded shown in Additional File 4. Figure 14 illustrates the distribution of reviews between 1988 and 2024. Those related to increasing SOC are the most common and are distributed throughout the period (*n* = 48), 18 of which were published between 2020 and 2023. Some reviews included multiple intervention groups, which were separated, resulting in a total of 91 entries.

#### Review type

**Fig. 15:**
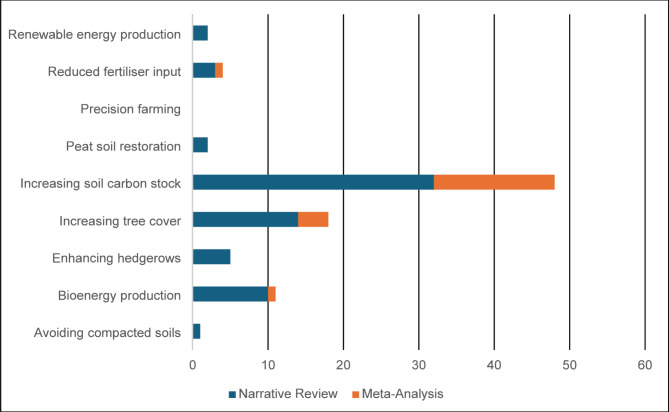
Number of reviews by type

There were 69 narrative reviews, 22 meta-analysis and no systematic reviews (Fig. 15). A narrative review was defined as a review where synthesis was carried out without combining data. Meta-analyses were defined as reviews that included combining data alongside a narrative review. Systematic reviews were defined as reviews that had published a protocol including a defined and replicable search strategy prior to the search being conducted.

#### Population and land management objective reviews

**Fig. 16:**
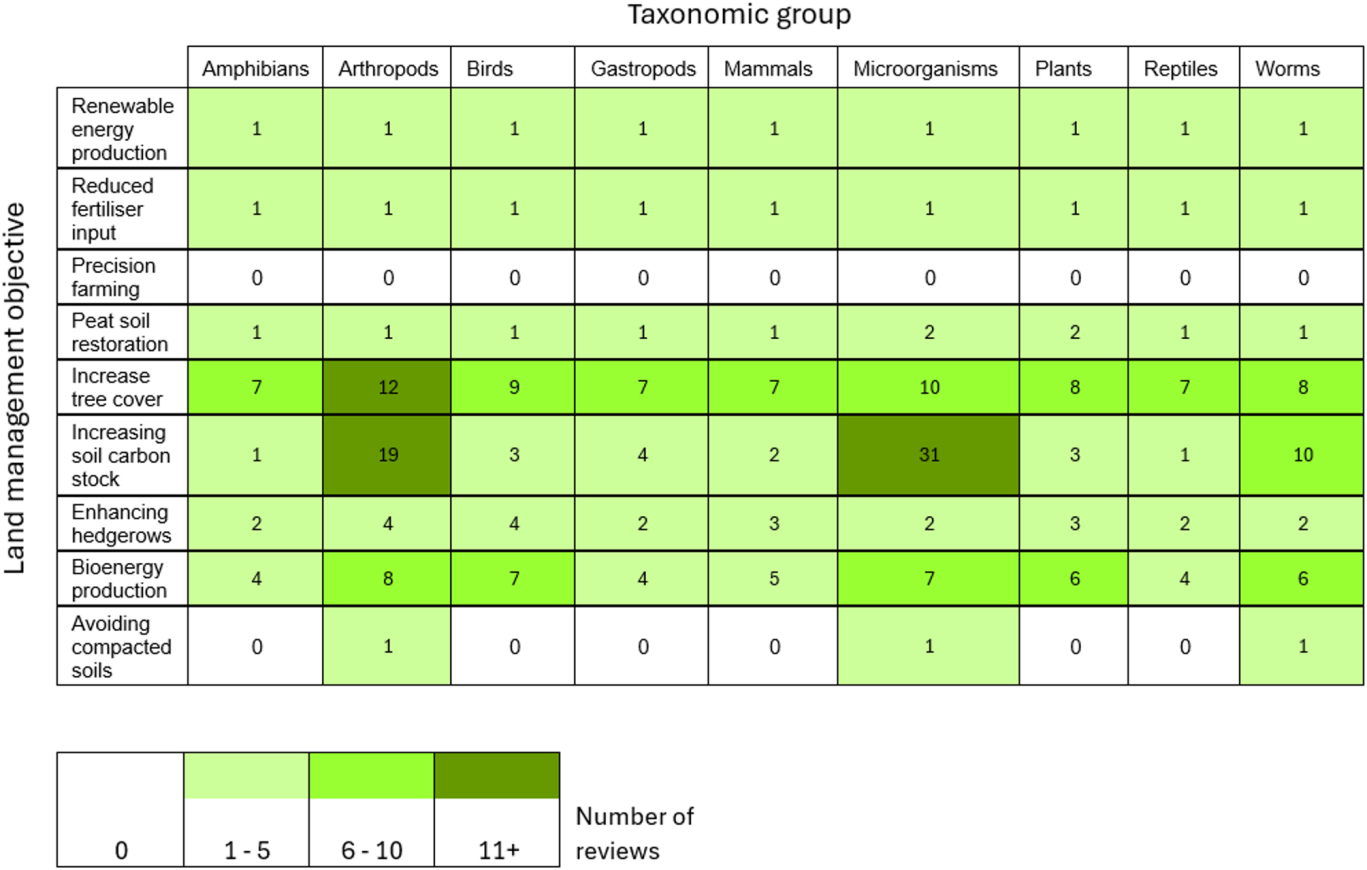
Heatmap of land management objectives and population group for reviews

The distribution of reviews in the heatmap (Fig. 16) showing the measured population against the land management objective largely matches the distribution of the primary evidence base. Most reviews focused on interventions that increase SOC and their impact on belowground biodiversity, such as microorganisms (*n* = 31), arthropods (*n* = 19) and worms (*n* = 10). Many reviews included papers on multiple taxonomic groups; therefore, the sum of this table is greater than the total number of included reviews.

**Fig. 17:**
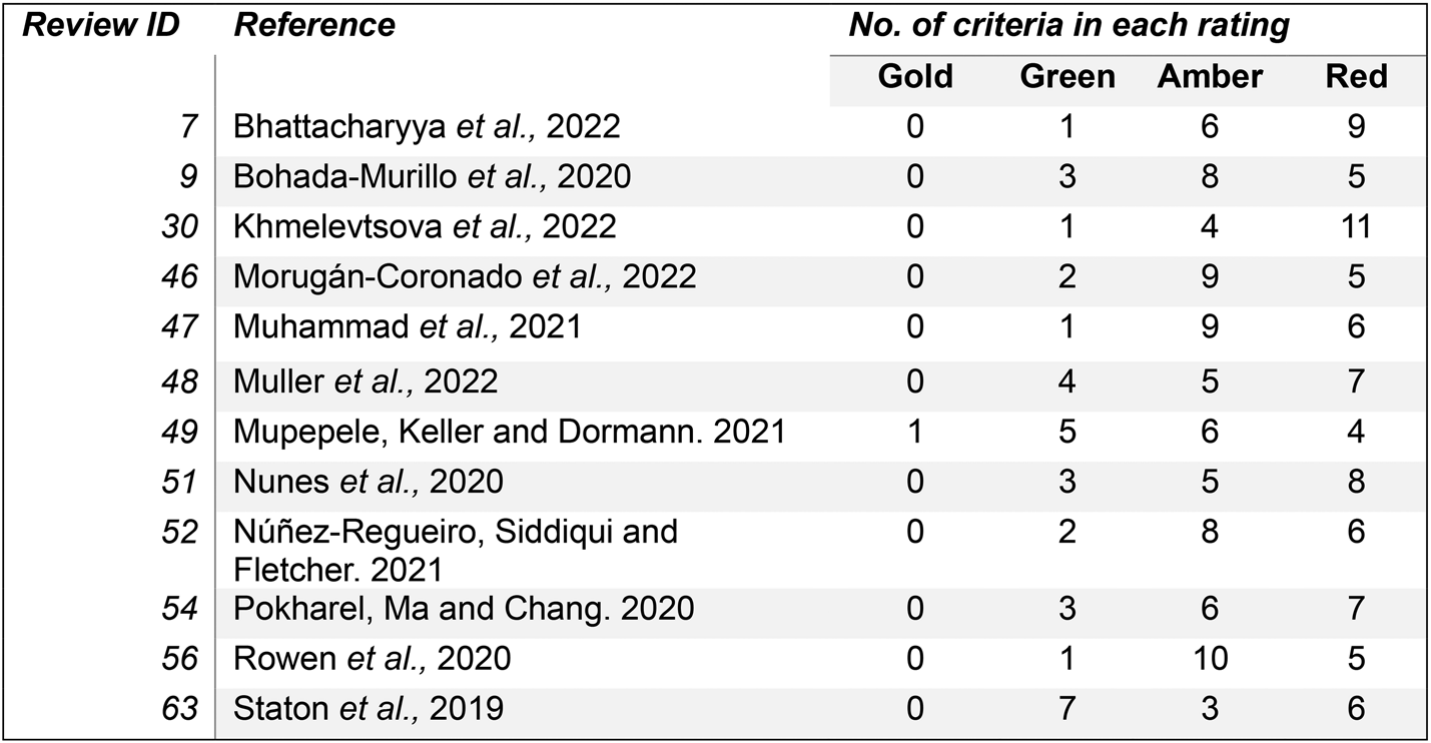
Results for reviews in the evidence base that are displayed on CEEDER. There are a total of 16 criteria, each one is rated on a colour scheme. Gold is the highest score, red is the lowest. Number relates to how many times each score was awarded

The reliability score for 12 reviews that have been assessed by CEESAT [44], are summarised in Fig. 17. Only one review received a gold rating (for reporting the number and titles of excluded articles during screening), most scores for all reviews were either amber or red. Notably, no review referenced a predetermined methodology or protocol and only one review provided elements of validity assessments for included articles. Thus, indicating low methodological rigour and a potential for bias in the evidence synthesis of this subset of reviews.

### Limitations of the map

A limitation of the map was that it only included publications written in or translated within a database to English. This is may have biased the distribution of the included papers to publications from English-speaking countries. Although few articles were excluded for this reason (*n* = 3).

Although a large subsection of articles was screened by two people (*n* = 850), only one person (SR) screened the remaining manually screened articles, and not all articles were screened due to the volume of articles obtained by the searches. Instead, the priority screening function of EPPI-Reviewer-Web was employed, and screening terminated when the inclusion rate at title and abstract level, was less than 1%. Although it is likely that the pool of unscreened articles was unlikely to contain any articles that meet the inclusion criteria, there is a chance that a small number of relevant articles may have been excluded.

Finally, where there was missing information during coding, the article’s authors were not contacted. Similarly, eligible articles for which the full text was not available were either coded at the abstract level or excluded if there was not sufficient detail in the abstract to warrant inclusion.

The subjects of land management, technology, policies and incentives to reduce the agricultural carbon footprint are developing at pace. The interventions included within this map were those promoted by the NFU and UK CCC. Other stakeholders, such as industry and non-governmental organisations, may promote interventions not included in this map, for example, dietary change (reducing livestock numbers) or introducing herbal leys to a rotation to reduce reliance on synthetic nitrogen fertilisers [48]. This is a limitation of this evidence base, but the search string, can be adapted to include/exclude interventions.

## Conclusions

This systematic map revealed evidence relating to how carbon footprint-reducing interventions impact farm biodiversity. Three databases and nine associated organisational websites were searched. The methodology endeavoured to reduce the introduction of bias by following CEE guidance [39], having published a protocol with a predetermined search and coding strategy [41]. The search returned 67,607 articles, which, after duplicate removal and screening processes, generated an evidence base of 820 studies.

### Implications for research

Global environmental targets are becoming increasingly at the forefront of government policy and business management; therefore, this area of research is likely to continue moving at pace. This evidence base should be updated every 2 to 3 years to inform research and policy, including refreshing the intervention list if necessary.

The evidence base should be used as a platform to launch systematic reviews and meta-analyses, where appropriate, on carbon footprint-reducing interventions to confirm whether its relationship with taxonomic groups or species is positive or negative and if there are trade-offs between [38]. Understanding an interventions impact on food production is also important [35]. Systematic reviews can provide a more robust and unbiased view of these relationships. This is lacking in the current review evidence base (Figs. 15 and 16).

The research question for this evidence map was broad and can be unpacked further in more specific evidence maps or systematic reviews with meta-analyses. For example, more detail can be extracted for individual interventions, such as comparisons of their establishment, management, agricultural productivity, soil type, topography, sample size, and experimental area, as well as critical analysis. Further research gaps and possible synergies could result from this.

Almost half of the evidence base included a control in its experimental design, indicating robust evidence. Although, it is not always possible to carry out ecology studies with a control, where possible, researchers should strive to do this to improve the robustness of their results [49].

Within the evidence base, 81% of the studies were conducted for less than 5 years (Fig. 9). Short studies (less than 1 year) were the most common in the evidence base. Long-term studies are needed for many interventions, such as tree planting, to understand their impact on biodiversity at different stages of its lifecycle. Multiyear studies are also needed to more confidently report evidence of change due to an intervention rather than an ecological shock such as abnormal weather or a disease outbreak [50].

There are many areas for further primary research, as shown by boxes with low numbers in the intervention group and the taxonomic group heatmap (Fig. 11). Experiments observing changes in the abundance of species often considered pests are likely to provide information that farmers need before altering agricultural production systems [51, 52]. This evidence base found that gastropods were observed only 16 times, and mammals 13 times which are often considered agricultural pests (such as slugs (e.g., *Arion ater*) and rabbits (e.g., *Oryctolagus cuniculus*).

Although individual study authors were more likely to indicate an increase in species abundance and/or diversity due to land management objectives to reduce the carbon footprint of agriculture, quality appraisal of individual research studies was either not carried out as part of the systematic mapping exercise or was very limited. We therefore recommend quality appraisal and further synthesis of the impacts of specific interventions or groups of interventions to confirm the findings indicated by the vote counting exercise.

The research question in this evidence review was designed to be relevant to the UK’s climate and agricultural production systems. Studies carried out in the UK were the most numerous (*n* = 150). However, many interventions have not been investigated on UK agricultural land or are underrepresented in the evidence base despite being promoted by various organisations (the UK government [53], the CCC [31] and the NFU [30]). Therefore, primary research is needed to determine whether a given intervention applied in the UK will produce similar outcomes for biodiversity to studies carried out elsewhere in the world. Due to the heterogeneity of UK landscapes, research may also need to be replicated across different UK landscapes. In the UK, fewer than ten studies per taxonomic group investigated changes to abundance and diversity of amphibians, gastropods, mammals, and reptiles. Many species within these taxonomic groups have a low impact on ecosystem services within the farmed environment (other than those considered pests), which may be a reason why they have not attracted research attention. To fully understand an intervention’s relationship with biodiversity for nature recovery purposes, these groups need to be studied.

### Implications for policy/management

This evidence map was designed to be used by policy setters and stakeholders who play a role in influencing temperate agricultural land management strategies designed to reach CNZ and nature recovery. The database of relevant publications allows those employing/promoting a carbon footprint-reducing intervention to base predictions as to how it might impact agriculturally associated biodiversity on a comprehensive evidence-base. This can be achieved by filtering the database by intervention and taxonomic group to display the relevant cluster of evidence and then summarising the author reported outcomes (e.g., how the evidence was presented for increasing SOC interventions in Fig. 13).

Policy makers should focus on commissioning research that will reduce knowledge gaps relating to interventions with no or little evidence for their impact on biodiversity. Projects should be funded to gather long-term datasets where possible to capture the full impact of interventions on biodiversity throughout its lifecycle. Specific to the UK, more research is required to investigate the impact of increasing tree cover and peatland restoration, given it is proposed that these be employed on large areas of UK agricultural land [31].

Policy makers also need to be aware of the methodology employed in evidence reviews and should commission methodologically rigorous evidence reviews. Reviews included in this evidence map that have been assessed by CEESAT are deemed to have low rigour in their methodology, since none of the reviews published a protocol or conducted thorough critical appraisal of their included evidence. Thus, there is potential for bias within them.

In countries with high population densities where agricultural land represents a high proportion of the non-urban land area, such as the UK, understanding how reducing the carbon footprint of agriculture impacts biodiversity, food production and energy production is a critical piece of the puzzle to meet global environmental targets [35]. This evidence base is intended to direct primary studies, and evidence reviews to further the understanding of this nexus of global land use challenges.

## Supplementary Information

Below is the link to the electronic supplementary material.

Supplementary Material 1.

Supplementary Material 2.

Supplementary Material 3.

Supplementary Material 4.

## Data Availability

No datasets were generated or analysed during the current study.
