# Correction: What evidence exists on how biodiversity is affected by the adoption of carbon footprint-reducing agricultural practices? A systematic map

**DOI:** 10.1186/s13750-025-00376-3

**Published:** 2025-12-13

**Authors:** Stuart Rowlands, Julia Casperd, Michael R. F. Lee, Scott Kirby, Nicola Randall

**Affiliations:** Harper Adams University, Edgmond, TF10 8NB Shropshire UK

**Correction: Environmental Evidence (2025) 14:16** 10.1186/s13750-025-00372-7

In this article [1], Fig. 17 appeared incorrectly and has now been corrected in the original publication. For completeness and transparency, the correct and incorrect versions are displayed below.

Incorrect Fig. 17

**Fig. 17:**
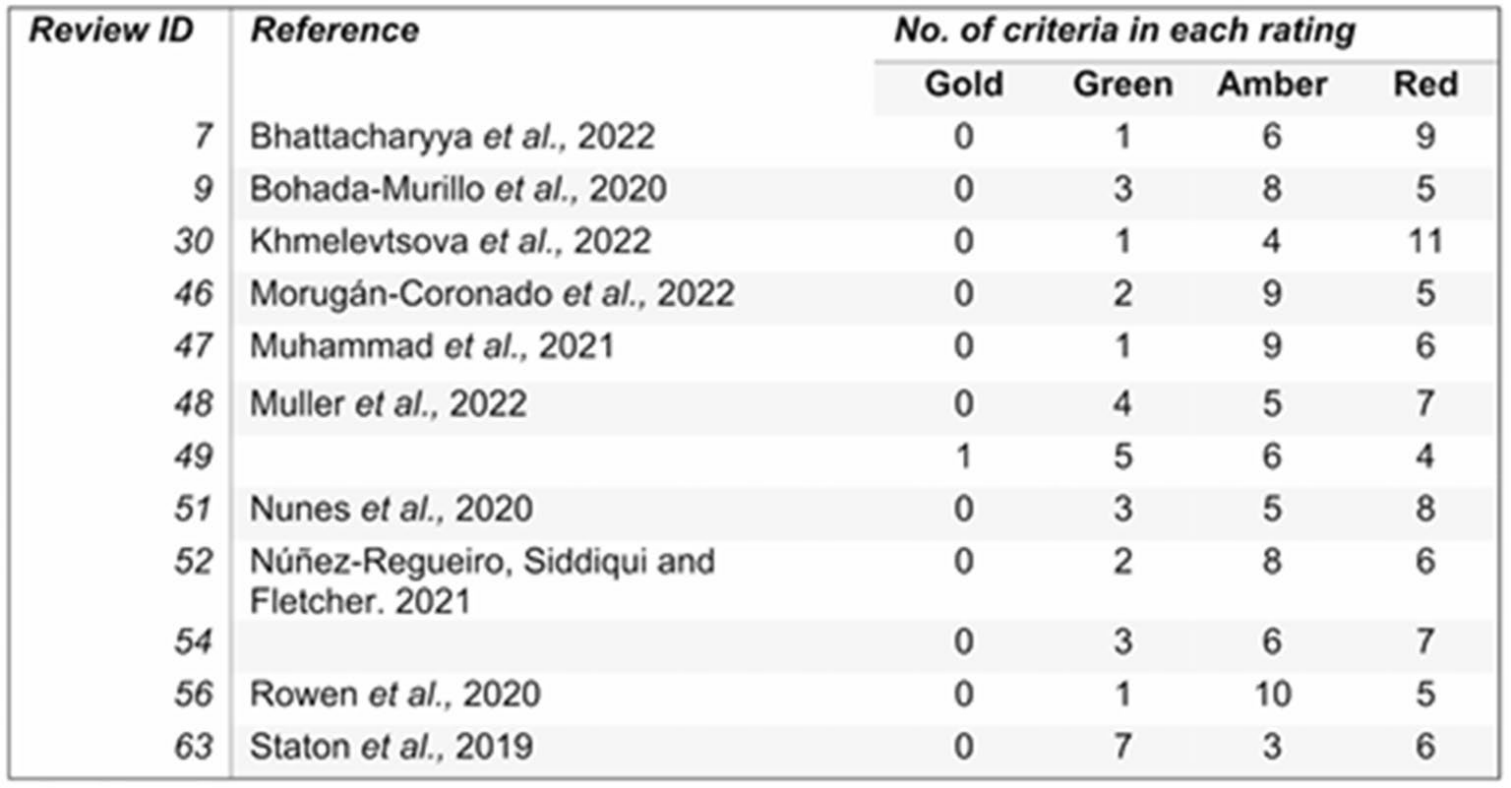
Results for reviews in the evidence base that are displayed on CEEDER. There are a total of 16 criteria, each one is rated on a colour scheme. Gold is the highest score, red is the lowest. Number relates to how many times each score was awarded

Correct Fig. 17

**Fig. 17:**
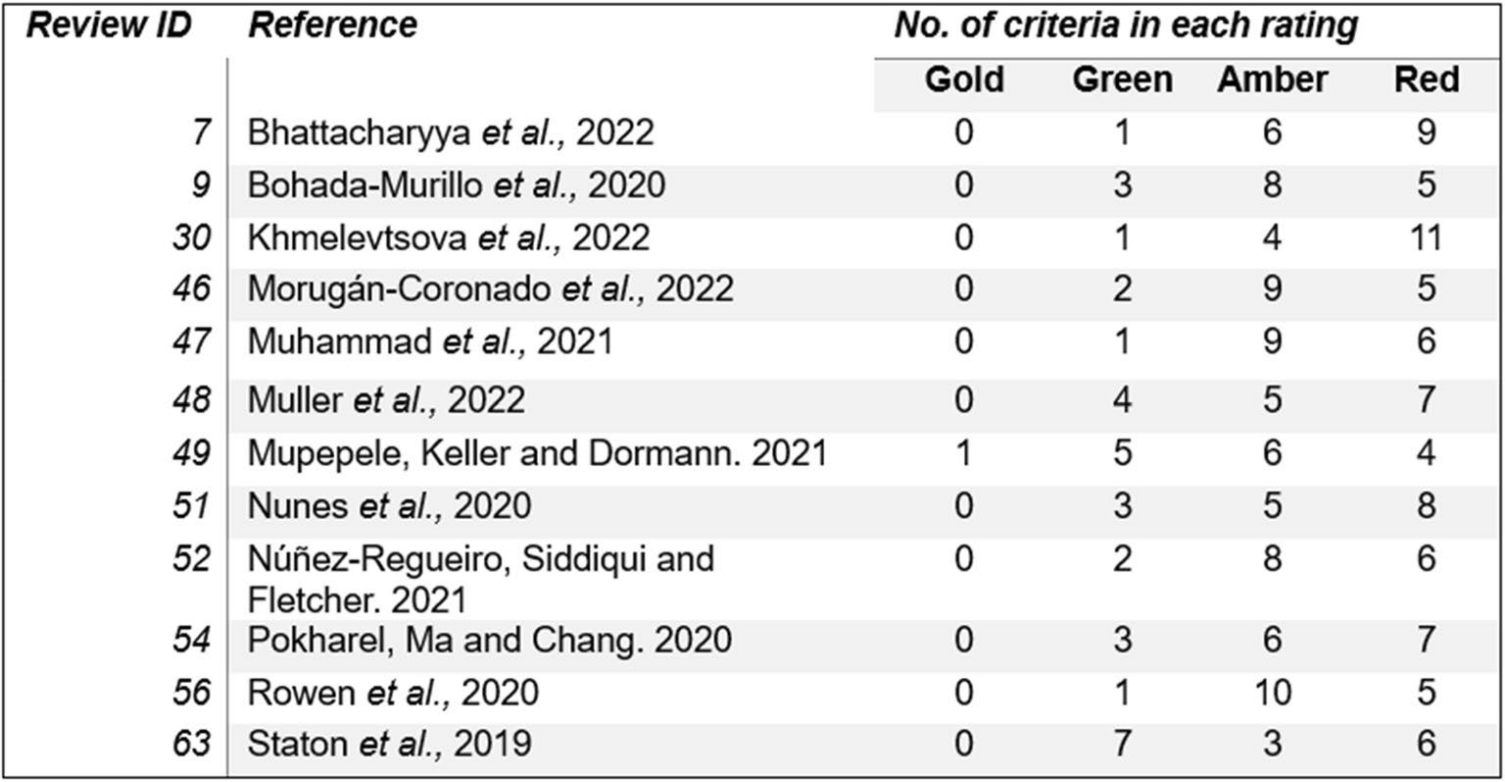
Results for reviews in the evidence base that are displayed on CEEDER. There are a total of 16 criteria, each one is rated on a colour scheme. Gold is the highest score, red is the lowest. Number relates to how many times each score was awarded

The original article has been corrected.
